# The new allosteric inhibitor asciminib is susceptible to resistance mediated by ABCB1 and ABCG2 overexpression *in vitro*

**DOI:** 10.18632/oncotarget.24393

**Published:** 2018-02-03

**Authors:** Laura N. Eadie, Verity A. Saunders, Susan Branford, Deborah L. White, Timothy P. Hughes

**Affiliations:** ^1^ Cancer Theme, South Australian Health and Medical Research Institute (SAHMRI), Adelaide, South Australia; ^2^ School of Medicine, University of Adelaide, Adelaide, South Australia; ^3^ School of Biological Sciences, University of Adelaide, Adelaide, South Australia; ^4^ Department of Genetics and Molecular Pathology, Centre for Cancer Biology, SA Pathology, Adelaide, South Australia; ^5^ School of Pharmacy and Medical Science, University of South Australia, Adelaide, South Australia; ^6^ School of Paediatrics, University of Adelaide, Adelaide, South Australia; ^7^ Division of Haematology, SA Pathology, Adelaide, South Australia

**Keywords:** asciminib, ABL001, ABCB1, ABCG2, resistance

## Abstract

Asciminib (previously ABL001), which binds the myristate-binding pocket of the Bcr-Abl kinase domain, is in phase I clinical trials as monotherapy and in combination with imatinib, nilotinib and dasatinib for the treatment of patients with refractory CML or Ph+ ALL. Asciminib sensitivity was evaluated in asciminib naïve *BCR-ABL1+* cell lines K562 (negligible ABCB1/ABCG2 expression), K562-Dox (ABCB1-overexpressing through doxorubicin exposure) and K562-ABCG2 (ABCG2 overexpression via transduction) with results demonstrating asciminib efflux by both ABCB1 and ABCG2 transporters. K562-Dox and K562-ABCG2 cells demonstrated increased LD50^asciminib^ vs K562 control cells: 256 and 299 nM respectively vs 24 nM, *p <* 0.001. Sensitivity was completely restored with specific inhibitors cyclosporine (ABCB1) and Ko143 (ABCG2): K562-Dox LD50^asciminib+cyclosporine^ = 13 nM, K562-ABCG2 LD50^asciminib+Ko143^ = 15 nM (*p* < 0.001). When asciminib resistance was modelled *in vitro*, ABCB1 and ABCG2 overexpression was integral in the development of asciminib resistance. In K562 asciminib-resistant cells, ABCG2 expression increased prior to *BCR-ABL1* overexpression and remained high (up to 7.6-fold greater levels in resistant vs control cells, *p <* 0.001). K562-Dox asciminib-resistant cells had increased ABCB1 expression (2.1-fold vs control cells *p* = 0.0033). KU812 asciminib-resistant cells overexpressed ABCB1 (5.4-fold increase, *p <* 0.001) and ABCG2 (6-fold increase, *p <* 0.001) before emergence of a novel myristate-binding pocket mutation (F497L). In all three cell lines, asciminib resistance was reversible upon chemical inhibition of ABCB1, ABCG2 or both (*p <* 0.001). When K562 asciminib-resistant cells were treated with asciminib in combination with clinically achievable doses of either imatinib or nilotinib, reversal of the resistance phenotype was also observed (*p <* 0.01). Overexpression of efflux transporters will likely be an important pathway for asciminib resistance in the clinical setting. Given the lack of evidence for ABCG2-mediated transport of nilotinib or imatinib at clinically relevant concentrations, our data provide an additional rationale for using asciminib in combination with either TKI.

## INTRODUCTION

The first generation ATP-competitive tyrosine kinase inhibitor (TKI) imatinib was designed to bind the ATP-pocket of Bcr-Abl [1]. Imatinib, and the second generation inhibitors nilotinib and dasatinib, have resulted in excellent overall and event free survival rates in chronic myeloid leukemia (CML) patients [2–4]. However, discontinuation of imatinib due to intolerance or resistance is still a significant problem in up to 35% of patients [5, 6] and more recently, resistance to nilotinib and dasatinib has also been observed [3, 4, 7]. The most common mechanism of acquired resistance is point mutations in the *BCR-ABL1* kinase domain [8], including development of the ‘gatekeeper’ T315I mutation [9–11]. T315I demonstrates resistance to all first and second generation inhibitors [12, 13], and the frequency of development increases with disease progression and exposure to multiple TKIs. While the third generation inhibitor ponatinib demonstrates activity against cells harboring the T315I mutation *in vitro* [14] and is successful at reducing disease burden *in vivo* [15, 16], it is associated with significant safety concerns [17].

The new allosteric inhibitor, asciminib (previously ABL001), belongs to a class of drugs designed to inhibit Bcr-Abl by binding to a distinct and separate region of the kinase domain from that where ATP-competitive TKIs bind: the myristate-binding pocket [18, 19]. Native c-Abl1 contains a myristate moiety that functions as an auto regulator; however, the myristate group is lost upon fusion to Bcr causing the constitutive activation associated with Bcr-Abl [20, 21]. Asciminib and other allosteric inhibitors mimic the myristate group locking Bcr-Abl in an inactive conformation and inhibiting kinase activity [22, 23]. Following preclinical modelling, which demonstrated both sustained elimination of tumors in a mouse model of leukemia (when used in combination with nilotinib) and activity against clinically relevant kinase domain mutations *in vitro* [23], asciminib entered open label phase I clinical trial for patients with refractory CML or Ph+ ALL (http://clinicaltrials.gov/show/NCT02081378) alone and in combination with imatinib or nilotinb or dasatinib.

In this study three asciminib resistant cell lines were generated as a means of modelling resistance in patients. Given that asciminib is currently administered to previously treated patients likely harboring resistance mechanisms known to develop in response to ATP-competitive TKIs, resistant cell lines were interrogated for *BCR-ABL1* overexpression, aberrant activation of proteins involved in kinase signalling pathways (eg: p-STAT3, p-STAT5A/B) and presence of kinase domain mutations. Data from our laboratory and others have demonstrated overexpression of the drug efflux transporters ABCB1 and ABCG2 are key in initiation of resistance to TKI therapy [24–27]. We have also recently highlighted the potential importance of monitoring ABCB1 expression levels in order to predict outcome to TKI therapy [28]. Thus, asciminib sensitivity was also evaluated in the setting of ABCB1 and ABCG2 overexpression. Results from both experimental arms demonstrate that asciminib is susceptible to resistance mediated by ABCB1 and ABCG2 overexpression. Importantly, the concomitant use of ABCB1 and ABCG2 inhibitors or imatinib/nilotinib reversed the observed resistance suggesting combination treatment approaches are justified.

## RESULTS

### Asciminib is transported by both ABCB1 and ABCG2

**Figure 1:**
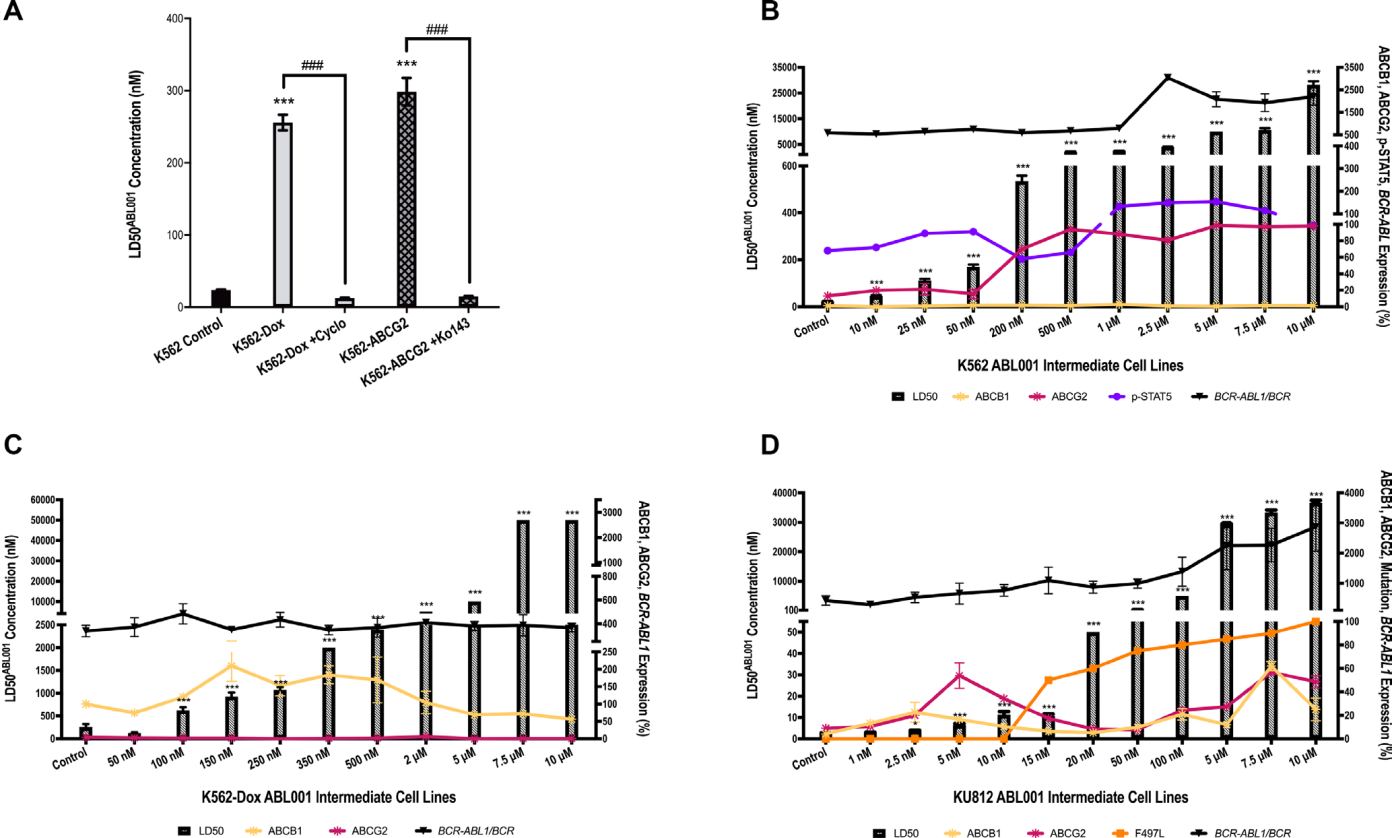
Asciminib is susceptible to resistance mediated by overexpression of the drug efflux transporters ABCB1 and ABCG2 (**A**) K562, K562-Dox and K562-ABCG2 cells were cultured for 72 h in increasing concentrations of asciminib in the absence and presence of the ABCB1 inhibitor cyclosporine (cyclo) and the ABCG2 inhibitor Ko143. The concentration of asciminib required to kill 50% of cells (LD50^asciminib^) was determined by Annexin V/7-AAD staining. (**B**) K562 (**C**) K562-Dox (**D**) KU812 cells were cultured long-term in gradually increasing concentrations of asciminib and resistance determined by LD50^asciminib^ (hatched bars). Resistance intermediates were also assessed for expression levels of ABCB1 and ABCG2 protein by flow cytometry (yellow and maroon lines respectively). The percentage of the cell population positive for transporter expression was normalised to individual cell line isotype controls and the resultant percentage positivity reported. p-STAT5 levels were assessed by the Milliplex^®^ MAP assay (purple line, expressed as MFI), *BCR-ABL1* mRNA was evaluated (black line, expressed as ratio of BCR) and the kinase domain sequenced for mutations (orange line). With the exception of the Milliplex^®^ MAP assay and sequencing which were performed once, data represent the mean of at least three experiments. Analyses were performed using unpaired Student’s *t*-test (Welch’s correction was applied for data groups with unequal SD) or Mann-Whitney Rank Sum test. Statistically significant increases in LD50^asciminib^ compared with control are denoted by asterisks; significant decreases in the presence of ABCB1/ABCG2 inhibitors are denoted by hashes (^***^p < 0.001). Error bars represent SEM.

Preclinical modeling in Ba/F3 cells [23, 29] indicates that asciminib is transported by ABCB1, however, we now show that asciminib is also transported by ABCG2. Asciminib-mediated cell death was evaluated in K562-Dox (ABCB1 overexpressing) and K562-ABCG2 overexpressing cells compared with parental K562 cells (negligible ABCB1 and ABCG2 expression). Results demonstrate a significant increase in LD50^asciminib^ in both cell lines: K562 LD50^asciminib^ = 24 nM vs K562-Dox LD50^asciminib^ = 256 nM (*p <* 0.001) and K562-ABCG2 LD50^asciminib^ = 299 nM (*p <* 0.001). Importantly, sensitivity to asciminib was completely restored upon inhibition of ABCB1 and ABCG2 with inhibitors cyclosporine and Ko143 respectively: K562-Dox LD50^asciminib+cyclosporine^ = 13 nM (*p <* 0.001) and K562-ABCG2 LD50^asciminib+Ko143^ = 15 nM (*p <* 0.001; Figure 1A).

### K562 asciminib resistant cells express sustained high levels of ABCG2

The current clinical trial protocol designates use of asciminib in patients who are relapsed or refractory to, or who are intolerant of, TKIs. In order to recapitulate this *in vivo* situation, common resistance mechanisms observed in response to TKI therapy were assessed for ability to confer resistance to asciminib *in vitro*. Possible resistance mechanisms that may develop as a result of asciminib therapy were also investigated (TKI intolerant patients). In this study, resistance to asciminib was generated in three *BCR-ABL1*+ cell lines: K562, K562-Dox and KU812 as previously described [30]. Resistance to asciminib was evaluated at every dose escalation intermediate by cell viability assays and common mechanisms of resistance interrogated. Because of the likely involvement of efflux transporters [31], particular focus was given to monitoring the expression levels of ABCB1 and ABCG2. Indeed, results indicated overexpression of one or both transporters was integral in development of resistance to asciminib in all cell lines investigated.

**Figure 2:**
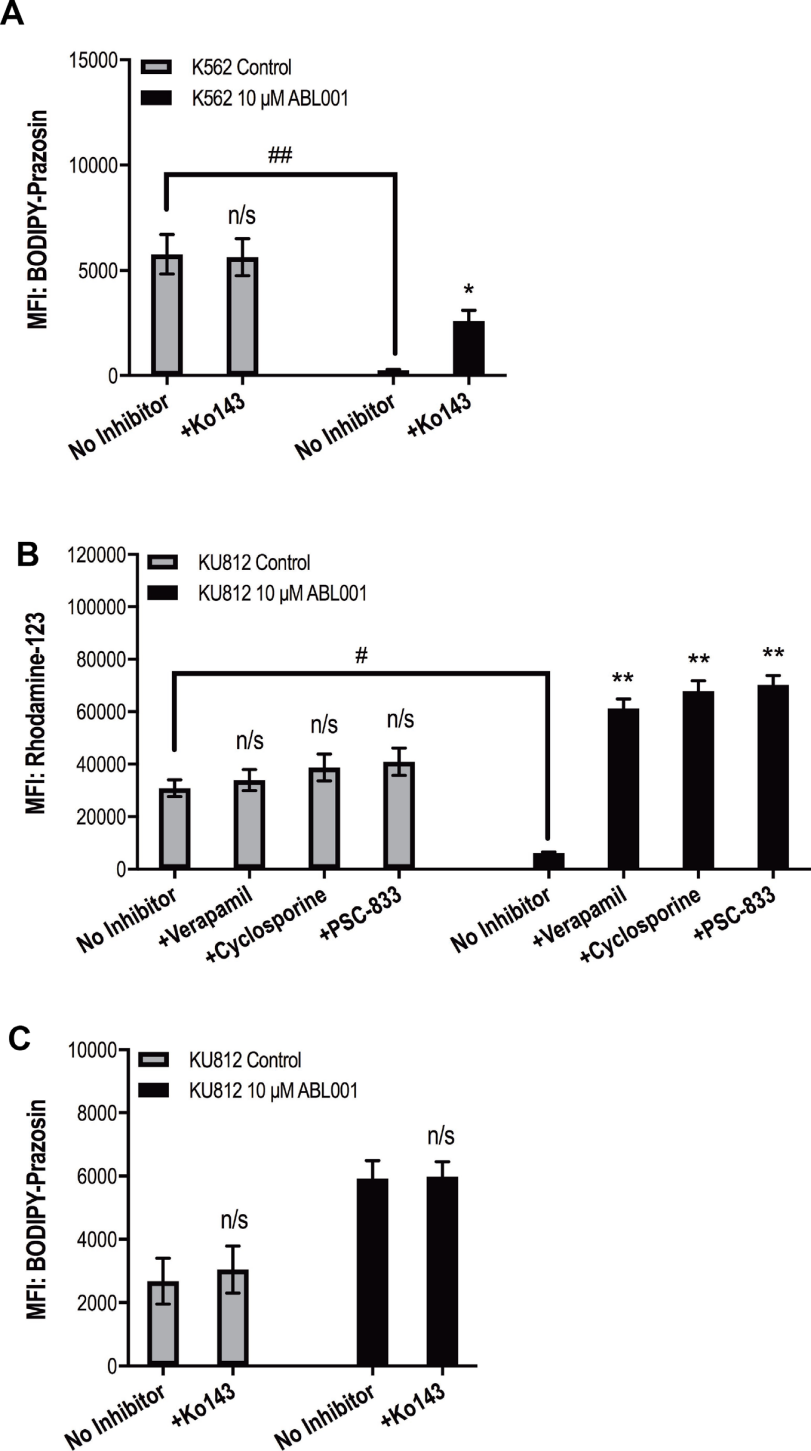
Increased function of ABCB1 and ABCG2 is responsible for asciminib resistance (**A**) K562 and (**B–C**) KU812 cells were stained with the fluorescent substrates BODIPY–prazosin and rhodamine 123 as indicated. Fluorescence was determined in the absence and presence of (A–B) the specific ABCG2 inhibitor Ko143 and (C) the ABCB1 inhibitors verapamil, cyclosporine and PSC-833. Data represent the mean of at least three experiments. Analyses were performed using unpaired Student’s *t*-test (Welch’s correction was applied for data groups with unequal SD). Statistically significant increases in MFI in the presence of transporter inhibition are denoted by asterisks; significant decreases in MFI in resistant cells compared with control cells are denoted by hashes (^*^p < 0.05, ^**^p < 0.01). Error bars represent SEM. MFI = mean fluorescent intensity. n/s = not significant.

Asciminib resistant K562 cells demonstrated a concordant increase in LD50^asciminib^ with increasing concentrations of asciminib (Figure 1B, *p <* 0.001). Interrogation of the mechanisms of resistance revealed an increase in ABCG2 expression in K562 200 nM asciminib cells (up to 7.6-fold greater levels in resistant cells compared with control cells, *p <* 0.001; Figure 1B) that remained high for the duration of asciminib dose escalation indicating the likely role of this transporter in asciminib resistance. Importantly, the ABCG2 present was functionally active providing resistant cells an increased ability to efflux the model ABCG2 substrate BODIPY-prazosin when compared with control cells. K562 10 μM asciminib cells demonstrated decreased levels of BODIPY-prazosin compared with K562 control cells (MFI = 251 vs 5765 respectively, *p <* 0.01, Figure 2A). In K562 10 μM asciminib cells, efflux of BODIPY-prazosin was inhibitable upon addition of the ABCG2 inhibitor Ko143 (MFI = 2575, *p =* 0.021, Figure 2A). In contrast, Ko143 had no effect on BODIPY-prazosin levels in K562 control cells (MFI = 5627, *p =* 0.919, Figure 2A). Late stage resistance intermediates also demonstrated elevated levels of *BCR-ABL1* and p-STAT5 (Figure 1B), two mechanisms of resistance previously observed in patients in response to imatinib therapy [32–35]. Investigation of Bcr-Abl protein expression levels demonstrated an increase in both total Bcr-Abl and phosphorylated Bcr-Abl (Y177, Y245; Supplementary Figure 1A). No overexpression of ABCB1 was observed and no mutations to the kinase or myristate binding domains were detected (data not shown). Taken together these data demonstrate increased expression of ABCG2 is the dominant mechanism of asciminib resistance in K562 cells.

### Asciminib resistance in K562-Dox cells is initiated by ABCB1 overexpression

Again, when resistance was assessed in each of the K562-Dox asciminib intermediates, a step-wise increase in LD50^asciminib^ was observed; LD50^asciminib^ in K562-Dox control cells was 256 nM, however negligible cell death occurred in the presence of 50 μM asciminib in K562-Dox 10 μM asciminib cells (*p <* 0.001, Figure 1C). K562-Dox cells already express high levels of ABCB1 however, upon exposure to asciminib, expression further increased to a maximum of 2.1-fold that observed in K562-Dox control cells (*p =* 0.0033, Figure 1C). No *BCR-ABL1* mRNA overexpression was observed (Figure 1C) and there was also no increase in total Bcr-Abl protein or phosphorylation of Bcr-Abl at Y177 or Y245 (Supplementary Figure 1B). No expression of ABCG2 was observed (Figure 1C), no kinase domain or myristate binding domain mutations developed and there were no alterations in STAT5 phosphorylation (data not shown).

### KU812 asciminib resistant cells demonstrate overexpression of both ABCB1 and ABCG2

Long-term culture of KU812 cells in increasing concentrations of asciminib resulted in a concordant increase in LD50^asciminib^ from 2.7 nM in KU812 control cells to 36 600 nM in KU812 10 μM asciminib cells (*p <* 0.001, Figure 1D). Asciminib exposure was accompanied by an immediate increase in expression of both ABCB1 and ABCG2. When compared with KU812 control cells, KU812 5 nM asciminib cells demonstrated significantly increased levels of ABCB1 (from 4 to 17%, *p <* 0.001) and ABCG2 (from 9 to 54%, *p <* 0.001; Figure 1D). Continued exposure of these cells to nanomolar concentrations of asciminib resulted in a reduction in ABCB1 and ABCG2 expression to basal levels and the concomitant emergence of the novel myristate binding domain mutation F497L (initially at 50%, later 100%, Figure 1D). Interestingly, upon sustained exposure to asciminib (concentrations >5 μM), expression levels of both transporters increased again suggesting the protection afforded by either/both transporters is necessary for high concentrations of asciminib. Indeed, assessment of the functional activity of ABCB1 in KU812 10 μM asciminib cells demonstrated decreased levels of rhodamine-123 compared with KU812 control cells (MFI = 6112 vs 30836 respectively, *p =* 0.015, Figure 2B). In KU812 10 μM asciminib cells, efflux of rhodamine-123 was reversible upon addition of all ABCB1 inhibitors tested indicating ABCB1 was functionally active: in the presence of verapamil, MFI = 61282 (*p =* 0.0039); cyclosporine, MFI = 67864 (*p =* 0.0035); PSC-833, MFI = 70129 (*p =* 0.0029). Conversely, no decrease in rhodamine-123 levels in the presence of any inhibitor was observed in KU812 control cells (Figure 2B). Unexpectedly, Ko143 had no effect on BODIPY-prazosin levels in KU812 10 μM asciminib cells (MFI = 5919 vs 5982, *p =* 0.937, in the absence vs presence of Ko143 respectively, Figure 2C), suggesting that while ABCG2 expression is increased in this late stage resistance intermediate, the transporter has limited functional activity.

*BCR-ABL1* mRNA expression increased steadily over the duration of asciminib resistance development with levels in KU812 10 μM asciminib cells 6.8-fold greater than levels in KU812 control cells (Figure 1D); determination of total Bcr-Abl protein expression confirmed these data and indicated an increase in phosphorylation at Y177 and Y245 (Supplementary Figure 1C). Taken together, these data suggest early overexpression of ABCB1 and ABCG2 facilitated emergence of the previously un-described asciminib resistant mutation F497L, and later overexpression of *BCR-ABL1*.

### Inhibition of ABCB1 and ABCG2 reverses asciminib resistance *in vitro*

**Figure 3:**
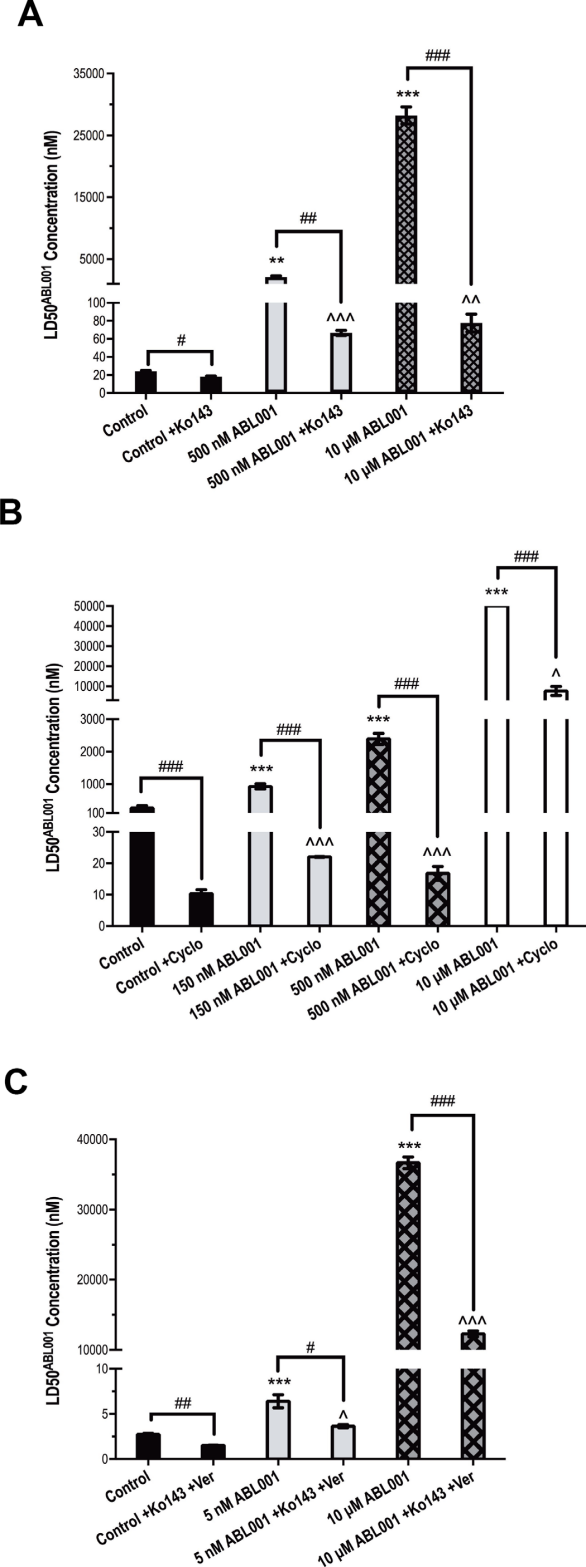
Inhibition of ABCB1 and ABCG2 reverses asciminib resistance *in vitro* LD50^asciminib^ was determined in (**A**) K562 control and 500 nM, 10 μM asciminib cells in the absence and presence of the ABCG2 inhibitor Ko143 (**B**) K562-Dox control and 150 nM, 500 nM, 10 μM asciminib cells in the absence and presence of the ABCB1 inhibitor cyclosporine (**C**) KU812 control and 5 nM, 10 μM asciminib cells in the absence and presence of Ko143 and verapamil. Statistical analyses compared 1) control cells vs resistant cells in the absence of inhibition (asterisks) 2) cells in the absence vs presence of ABCB1/ABCG2 inhibition (hashes) and 3) control cells in the absence of inhibition vs resistant cells in the presence of ABCB1/ABCG2 inhibition (carets). Analyses were performed using unpaired Student’s *t*-test (Welch’s correction was applied for data groups with unequal SD) or Mann-Whitney Rank Sum test. Statistically significant *p*-values are denoted by carets (^), hashes (#) or asterisks (^*^p < 0.05, ^**^p < 0.01, ^***^p < 0.001). Error bars represent SEM. Cyclo = cyclosporine; Ver = verapamil.

ABCB1 and/or ABCG2 overexpression was observed as a common mechanism of resistance to asciminib in all three *BCR-ABL1+* cell lines in this study. We subsequently assessed whether inhibition of these transporters could reverse asciminib resistance. Indeed, inhibition of ABCG2 in K562 500 nM asciminib cells (the resistance intermediate where 100% ABCG2 overexpression was first observed) with the specific inhibitor Ko143 resulted in a significant reduction in LD50^asciminib^ when compared with cells cultured in the absence of Ko143: 67 nM vs 2127 nM, *p =* 0.007; Figure 3A). Interestingly, even when Ko143 negated the effect of ABCG2 overexpression, K562 500 nM asciminib cells still exhibited a significantly increased LD50^asciminib^ compared with K562 control cells (24 nM vs 67 nM, *p <* 0.001; Figure 3A). In these resistant cells, p-STAT5 levels (MFI = 66) were similar to those observed in K562 control cells (MFI = 68); *BCR-ABL1* levels were slightly elevated (585 vs 667% *BCR-ABL1*/BCR, Figure 1B), although this increase failed to reach statistical significance. The same observations were made in K562 10 μM asciminib resistant cells: LD50^asciminib^ reduced from 28 200 nM to 78 nM (*p <* 0.001; Figure 3A) in the presence of Ko143. K562 10 μM asciminib resistant cells exhibit elevated levels of p-STAT5 (MFI = 99) compared with control as well as significantly increased levels of *BCR-ABL1* (585 vs 2196% *BCR-ABL1*/BCR, *p <* 0.001, Figure 1B). Taken together, these data indicate *BCR-ABL1* overexpression is sufficient to confer a degree of resistance to asciminib but that ABCG2 overexpression is the predominant mechanism of asciminib resistance in this cell line. As expected, there was a slight but significant decrease in LD50^asciminib^ in K562 control cells in the presence of Ko143 from 24 nM to 18 nM (*p =* 0.011, Figure 3A). This is likely due to the low basal level of ABCG2 expression (13%, Figure 1B).

Similarly, inhibition of ABCB1 with cyclosporine in K562-Dox cells caused a significant reduction in LD50^asciminib^ and reversal of asciminib resistance. K562-Dox 150 nM asciminib cells express 2.1-fold greater levels of ABCB1 when compared with K562-Dox control cells (Figure 1C) and exhibit a corresponding increase in LD50^asciminib^ (926 vs 256 nM, *p <* 0.001). This resistance is significantly reduced in the presence of cyclosporine to 22 nM (*p <* 0.001; Figure 3B). Analogous results were observed in K562-Dox 500 nM asciminib cells which express 1.7-fold greater levels of ABCB1 when compared with K562-Dox control cells (17 nM, *p <* 0.001; Figure 3B). As expected a significant reduction in LD50^asciminib^ in the presence of cyclosporine was also observed in K562-Dox control cells due to the high basal levels of ABCB1 expression in this asciminib naïve cell line: from 256 nM to 10 nM (*p <* 0.001; Figure 3B). Interestingly, when the LD50^asciminib^ values in the presence of cyclosporine were compared between control and resistant cells, the two resistant cell lines (150 nM and 500 nM asciminib) still had significantly increased LD50^asciminib^ values not attributable to ABCB1 expression indicating the presence of a second, as yet unidentified, resistance mechanism (*p <* 0.001).

Results from K562-Dox 10 μM asciminib cells confirmed this hypothesis; these cells express less ABCB1 than parental K562-Dox cells exhibiting a 1.8-fold decrease in ABCB1 expression. However, in the presence of cyclosporine, the LD50^asciminib^ remained elevated when compared with control cells in the absence of cyclosporine (7667 vs 256 nM, *p =* 0.030) and also when compared with the other resistance intermediates. These results suggest the secondary resistance mechanism is likely initially present in a smaller population of cells, which clonally expands over time given that no mutations were observed (data not shown) and *BCR-ABL1* levels do not significantly alter during the course of resistance generation (Figure 1C). In order to ascertain whether the resistance mechanism present was Bcr-Abl dependent or independent (whether asciminib could inhibit Bcr-Abl kinase activity by reducing auto-phosphorylation at Y245), phospho-Bcr-Abl protein levels were assessed in K562-Dox 10 μM asciminib cells that had been cultured continuously in asciminib compared with cells in which asciminib had been removed by thorough washing followed by overnight equilibration. Results demonstrated complete ablation of phospho-Bcr-Abl (Y245) in the presence of asciminib. However, upon drug washout, reactivation of Bcr-Abl kinase activity occurred resulting in similar levels of phospho-Bcr-Abl in both resistant and control cells (Supplementary Figure 2A). Importantly, total Bcr-Abl levels remained unaffected in the presence or absence of asciminib (Supplementary Figure 2B). Taken together, these results suggest the presence of a Bcr-Abl independent resistance mechanism.

KU812 5 nM asciminib resistant cells overexpress both ABCB1 and ABCG2 (4- and 6-fold increase respectively compared with control cells; *p <* 0.001, Figure 1D), thus we investigated the LD50^asciminib^ in the presence of dual ABCB1/ABCG2 inhibition. Cells were inhibited with verapamil and Ko143; verapamil was used in lieu of cyclosporine, which caused non-specific cytotoxicity due to the inherent sensitivity of this cell line whereas no significant cell death was observed in the presence of verapamil alone. The addition of both inhibitors significantly decreased LD50^asciminib^ from 6.4 to 3.6 nM (*p =* 0.021, Figure 3C). However, even with ABCB1/ABCG2 inhibition, KU812 5 nM asciminib cells maintained a significantly elevated LD50^asciminib^ compared with control cells (3.6 vs 2.7 nM, *p =* 0.034, Figure 3C) potentially due to *BCR-ABL1* overexpression (656% vs 426% in control cells, Figure 1D), although the meaningfulness of such a small increase is unlikely.

Similar results were observed in KU812 10 μM asciminib resistant cells, which also exhibit ABCB1 and ABCG2 overexpression (6- and 5.4-fold increase respectively compared with control cells; *p <* 0.001, Figure 1D). However, unlike KU812 5 nM asciminib cells, KU812 10 μM asciminib cells harbor the novel myristate-binding pocket mutation F497L (100%, Figure 1D) in addition to *BCR-ABL1* overexpression (2877% vs 426% in control cells, Figure 1D). Importantly, the majority of the resistance in asciminib-resistant KU812 cells is dependent on the overexpression of ABCB1 and ABCG2 as evidenced by LD50^asciminib^. In the absence of ABCB1/ABCG2 inhibition, KU812 10 μM asciminib LD50^asciminib^ = 36.7 μM (due to transporter overexpression, F497L mutation and *BCR-ABL1* overexpression). However, in the presence of verapamil and Ko143, KU812 10 μM asciminib LD50^asciminib^ = 12.3 μM (due to F497L mutation and *BCR-ABL1* overexpression only). This suggests that approximately double the amount of asciminib resistance is attributable to transporter overexpression (24.4 μM) than that due to mutation and *BCR-ABL1* overexpression combined (12.3 μM; Figure 3C).

### Cells harbouring the novel myristate-binding pocket mutation F497L are sensitive to imatinib and nilotinib

The F497L mutation was first detected at 50% in the KU812 15 nM asciminib intermediate and increased in percentage with every asciminib dose escalation up to 100% in KU812 10 μM asciminib cells (Figure 1D). F497L is a novel asciminib-resistant mutation and thus it was necessary to determine its resistance profile to other TKIs. While difficult to determine the exact sensitivity of KU812 10 μM asciminib cells to imatinib, nilotinib and dasatinib due to the multiple overlapping resistance mechanisms present in this cell line (F497L, ABCB1, ABCG2, *BCR-ABL1* overexpression; Figure 1D) we have evaluated TKI-sensitivity in the absence of transporter overexpression, which is responsible for the majority of asciminib resistance (Figure 3C).

**Figure 4:**
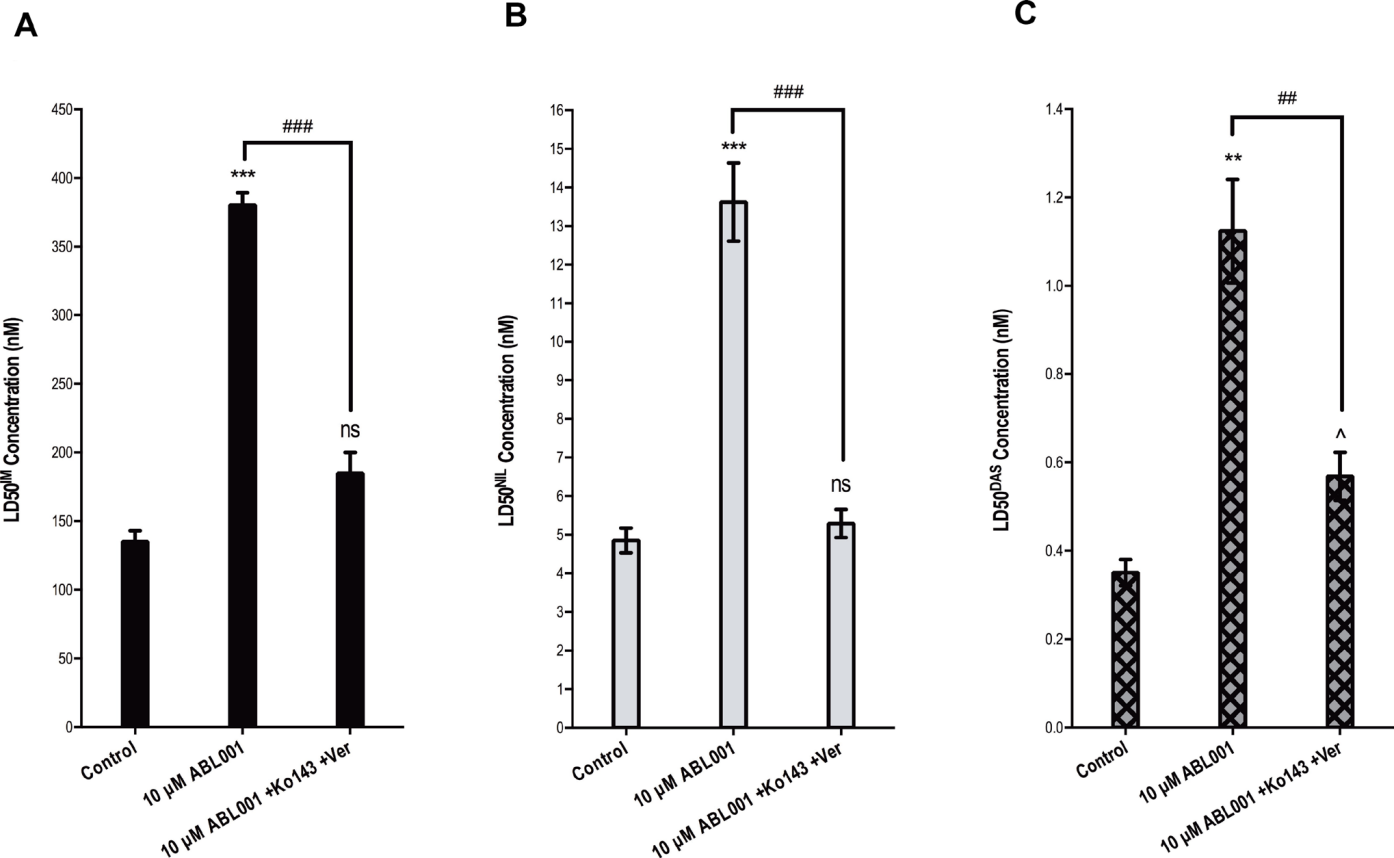
F497L demonstrates sensitivity to imatinib and nilotinib *in vitro* (**A**) LD50^IM^ (**B**) LD50^NIL^ and (**C**) LD50^DAS^ were determined in KU812 control cells compared with KU812 10 μM asciminib cells (F497L mutation at 100%) in the absence and presence of dual ABCB1 (verapamil) and ABCG2 (Ko143) inhibition. Statistical analyses compared 1) control cells vs resistant cells in the absence of inhibition (asterisks) 2) resistant cells in the absence vs presence of ABCB1/ABCG2 inhibition (hashes) and 3) control cells in the absence of inhibition vs resistant cells in the presence of ABCB1/ABCG2 inhibition (carets). Data represent the mean of at least three independent assays. Analyses were performed using unpaired Student’s *t*-test (Welch’s correction was applied for data groups with unequal SD). Statistically significant *p*-values are denoted by carets (^), hashes (#) or asterisks (^*^p < 0.05, ^**^p < 0.01, ^***^p < 0.001). Error bars represent SEM. Ver = verapamil.

ABCB1, ABCG2 and *BCR-ABL1* overexpression are previously defined resistance mechanisms to imatinib, nilotinib and dasatinib [24, 26, 30, 33] so, as expected, in the absence of ABCB1/ABCG2 inhibition, LD50^IM^, LD50^NIL^ and LD50^DAS^ were significantly increased in KU812 10 μM asciminib cells compared with control cells: LD50^IM^ increased from 135 to 380 nM (*p <* 0.001); LD50^NIL^ increased from 4.8 to 13.6 nM (*p <* 0.001); LD50^DAS^ increased from 0.35 to 1.1 nM (*p =* 0.002, Figure 4A–4C). Also as expected, in the presence of ABCB1/ABCG2 inhibition LD50^IM^ and LD50^NIL^ significantly decreased negating the resistance observed (*p <* 0.001). Conversely, while inhibiting ABCB1 and ABCG2 significantly decreased the LD50^DAS^ (*p <* 0.01) a significant level of dasatinib resistance remained (*p =* 0.035, Figure 4C). Taken together, these results suggest that cells expressing the F497L mutation are sensitive to imatinib and nilotinib but may cause some level of resistance to dasatinib. However, given that these cells are sensitive to clinically achievable doses of all three TKIs, F497L is unlikely to contribute to resistance in patients receiving TKIs.

### Asciminib used in combination with imatinib and nilotinib reverses resistance *in vitro*

Asciminib was developed for use in combination with ATP-competitive TKIs such as imatinib and nilotinib but is currently being trialed only in those patients who have failed one or more previous TKI therapies. Given that patients receiving asciminib for TKI resistance will likely harbor several resistance mechanisms, and as we have already demonstrated, there are mechanisms of resistance common to ATP-competitive TKIs and asciminib (overexpression of *BCR-ABL1*, ABCB1/ABCG2, p-STAT5) it is important to ascertain whether the use of nilotinib or imatinib in combination with asciminib increases asciminib efficacy in the resistant setting. While asciminib appears to be highly susceptible to ABCG2-mediated resistance (Figure 1A, 1B, 1D) ABCG2-mediated resistance to imatinib or nilotinib has not been consistently observed either *in vitro* or *in vivo* [31]. Furthermore, imatinib and nilotinib are likely to inhibit ABCG2 at clinically achievable concentrations [31]. Thus, determination of whether the addition of either of these TKIs could reverse resistance in K562 10 μM asciminib cells (ABCG2 and *BCR-ABL1* overexpression, Figure 1B) is critical. Concentrations of imatinib and nilotinib for use in these combination assays were selected based on cell death when they were used as single agents; a concentration that did not significantly affect cell death when used alone was required so that any potential synergistic effects could be observed. Concentrations of 150 nM nilotinib and both 1 μM and 2 μM imatinib were deemed satisfactory for use in resistant cells. However, because these concentrations caused significant cell death in control cells proportionately lower TKI concentrations were selected for evaluation of TKIs on asciminib efficacy (Supplementary Figure 3).

**Figure 5:**
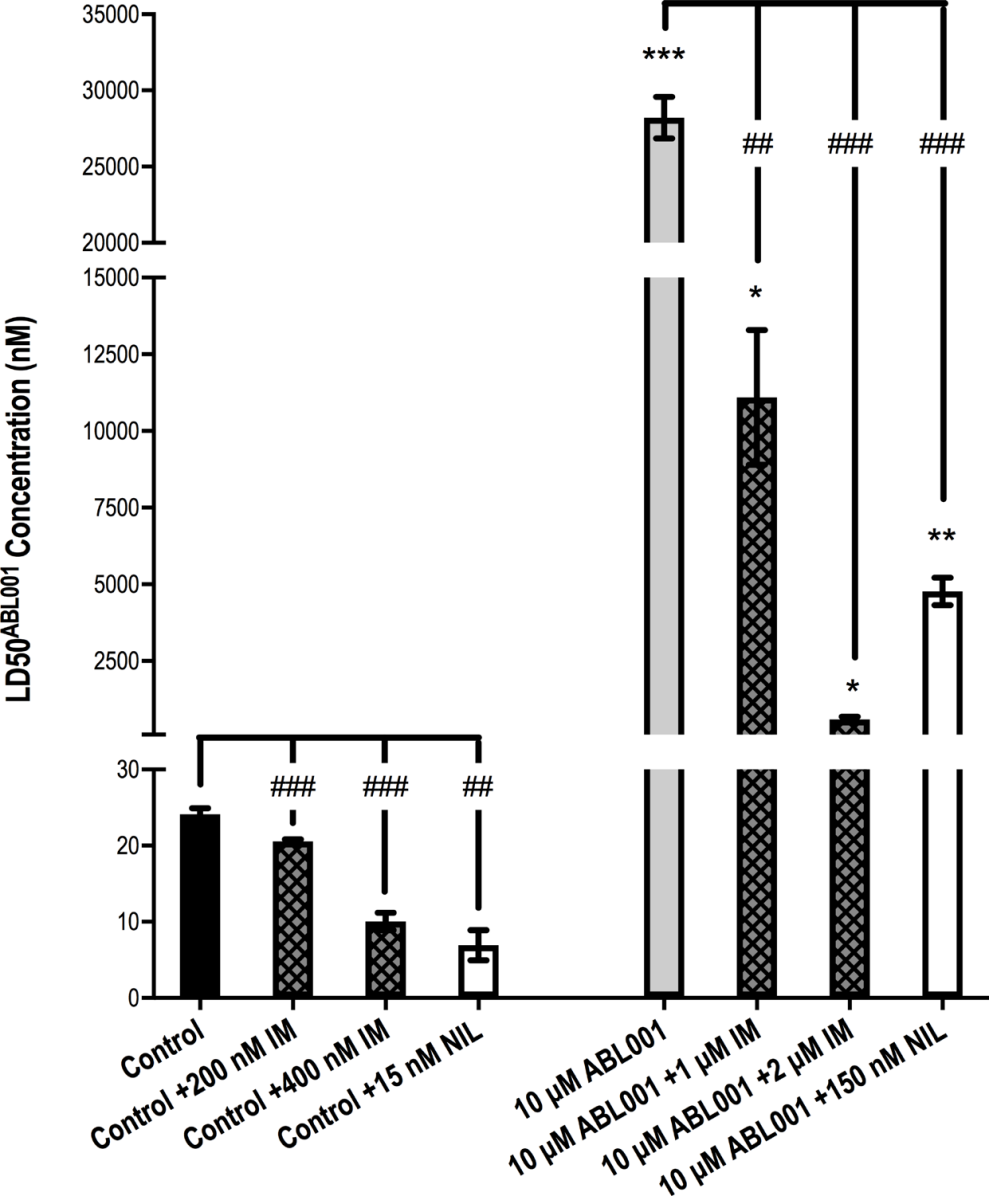
1 μM and 2 μM imatinib and 150 nM nilotinib significantly reverse resistance in K562 10 μM asciminib cells when used in combination with asciminib K562 10 μM asciminib cells were cultured for 72 h in increasing concentrations of asciminib in the absence and presence of 1 μM imatinib, 2 μM imatinib and 150 nM nilotinib. K562 control cells were cultured in proportionately less concentrations of TKI. The concentration of asciminib required to kill 50% of cells (LD50^asciminib^) was determined by Annexin V/7-AAD staining. Data represent the mean of at least 3 independent experiments. Analyses were performed using unpaired Student’s *t*-test (Welch’s correction was applied for data groups with unequal SD). Statistical analyses compared the LD50^asciminib^ in K562 10 μM asciminib vs K562 control cells (asterisks) and also the LD50^asciminib^ in K562 control/K562 10 μM asciminib when asciminib was used as a single agent vs asciminib used in combination with the specified concentrations of imatinib and nilotinib (hashes). Statistically significant alterations in LD50^asciminib^ are indicated (^*^p < 0.05, ^***^p < 0.01, ^***^p < 0.001). Error bars represent SEM. IM = imatinib. NIL = nilotinib.

As already described, K562 10 μM asciminib cells demonstrate a significantly increased LD50^asciminib^ (Figures 1B and 5), however, upon addition of 1 μM imatinib, LD50^asciminib^ was reduced from 28.2 μM to 11.1 μM (*p =* 0.004). Addition of 2 μM imatinib had a more dramatic effect resulting in a synergistic reduction in LD50^asciminib^ to 592 nM (*p <* 0.001, Figure 5; CI<1 for asciminib concentrations above 2500 nM, Supplementary Table 1). Similarly, addition of 150 nM nilotinib significantly reduced LD50^asciminib^ to 4.8 μM in an additive to synergistic manner (*p <* 0.001, Figure 5; Supplementary Table 1). These synergistic effects were also observed in K562 control cells: addition of 200 nM and 400 nM imatinib reduced LD50^asciminib^ from 24.1 nM to 20.6 nM (*p =* 0.0002, Figure 5; CI<1 for asciminib concentrations above 30 nM, Supplementary Table 1) and 10.0 nM (*p <* 0.0001, Figure 5; CI<1 for asciminib concentrations above 20 nM, Supplementary Table 1) respectively. The addition of 15 nM nilotinib also synergistically reduced the LD50^asciminib^ to 7.0 nM (*p =* 0.0012, Figure 5; CI<1 for asciminib concentrations above 25 nM, Supplementary Table 1). Given that imatinib and nilotinib, when used as single agents at the concentrations studied, had no significant impact on cell death (Supplementary Figure 3), these results provide the first evidence for use of two inhibitors in combination in the setting of transporter overexpression. Furthermore, patients treated with imatinib demonstrate steady-state plasma levels of between ∼2 and ∼5 μM (trough and peak levels, respectively) [36] while those patients treated with standard nilotinib therapy demonstrate steady-state plasma levels between ∼1.7 and ∼3.6 μM (trough and peak levels, respectively) [37] thus it would be anticipated that this effect would also occur in the clinical setting.

## DISCUSSION

We have demonstrated that asciminib is transported by both ABCB1 and ABCG2. Importantly, we have demonstrated that chemical inhibition of ABCB1 and ABCG2 increases asciminib efficacy leading to enhanced cell death *in vitro*. Preclinical *in vitro* studies into the efficacy of asciminib against various Bcr-Abl kinase domain mutations were performed in the mouse Ba/F3 cell line [23] whereas our current study evaluated the efficacy of asciminib in human *BCR-ABL1*+ cell lines. In addition we have generated three asciminib resistant cell lines and comprehensively interrogated them for mechanisms of resistance. We observed several recurring resistance mechanisms, many overlapping with those already known to cause, or which are indicators of, resistance to ATP-competitive TKIs: overexpression of *BCR-ABL1*, ABCB1/ABCG2, p-STAT5. We also identified a novel myristate-binding pocket mutation F497L located within the C-terminal of alpha helix 11 in the c-abl1 kinase domain (Genbank accession number: AAB60394.1). While F497 is positioned in the helix immediately adjacent to already identified asciminib resistant mutations (P465S, V468F) [23], the crystal structure of c-abl1 reveals the proximity is distal upon protein folding [38] (Supplementary Figure 4). The substitution of a phenylalanine residue for a leucine residue results in the loss of a benzene ring, which may have bearing on post-translational structure and interfere with asciminib binding, but this is speculation. Importantly, cells harbouring the F497L mutation are sensitive to clinically achievable concentrations of imatinib, nilotinib and dasatinib.

K562 asciminib resistant cells demonstrated an early increase in ABCG2 expression that remained for the duration of asciminib dose escalation; importantly, ABCG2 was functionally active. Interestingly, ABCB1 expression levels remained static throughout resistance development making it possible that asciminib has stronger binding affinity for ABCG2 compared with ABCB1 resulting in ABCG2 manifesting as the dominant asciminib transporter in K562 cells. An alternative explanation lies in the fact that K562 cells express low basal levels of ABCG2 but negligible levels of ABCB1 (mean = 13% and 1% respectively, Figure 1A). Thus, increased expression of ABCG2 was likely the most efficient route of resistance development for this cell line and no asciminib-mediated selection of ABCB1-overexpressing cells occurred. Overall, in our K562 cell line model of asciminib resistance, it is probable that exposure to asciminib increased ABCG2 overexpression. The subsequent reduction in intracellular asciminib concentrations then likely created a favourable environment for the increased Bcr-Abl activity and expression observed in later resistance intermediates. These results are confirmed by a recent study by Qiang *et al.* who also observed overexpression of ABCG2 in asciminib-resistant cell lines [39]. In contrast, KU812 asciminib resistant cells demonstrated increased expression of both ABCB1 and ABCG2, however only ABCB1 demonstrated the ability to effectively efflux a model substrate. There are two possible reasons for this: either the ABCG2 present in this cell line is functionally inactive or the level of ABCG2 expressed (24–59% at the time the efflux assays were performed) is not high enough for significant changes in BODIPY-prazosin levels to be observed and a more sensitive assay is required. The fact that KU812 cells express similar basal levels of each transporter (mean = 4% and 9% respectively) gives credence to the hypothesis that asciminib selects for the most efficient route of resistance development. It is likely that cells expressing higher levels of either ABCB1 and/or ABCG2 were equally likely to be favourably selected upon exposure to asciminib resulting in overexpression of both transporters as was observed in the bulk population.

Following continuous culture of K562-Dox cells in asciminib, ABCB1 levels decreased to 56.7% that observed in parental cells. This is the second time we have observed this reduction in ABCB1 levels in response to long-term culture in inhibitors of Bcr-Abl [27] which was unexpected given that these cells have stably expressed ABCB1 at 100% for over a decade. Intriguingly, an unidentified mechanism of resistance appears prevalent in later stage K562-Dox asciminib resistant cells. While asciminib was effective at inhibiting Bcr-Abl kinase activity in K562-Dox 10 μM asciminib cells, they remained completely unresponsive to the drug up to 50 μM; this resistance is not attributable to any of the usual candidates (*BCR-ABL1*/ABCB1/ABCG2 overexpression, increased activity of Bcr-Abl or STAT5, kinase domain mutations, myristate-binding pocket mutations). Instead, it is likely these cells harbor a novel Bcr-Abl independent mechanism of resistance such as activation of an alternative signaling pathway and investigations for the cause of resistance in this cell line are ongoing.

The combination of asciminib with imatinib and nilotinib resulted in significant reversal of resistance in K562 10 μM asciminib resistant cells. This could be due to TKI-mediated inhibition of the ABCG2 expressed in this intermediate. Our data demonstrate increasing reversal of resistance with increasing potency of TKI administered in combination: 1 μM imatinib < 150 nM nilotinib < 2 μM imatinib. However, similar observations were made in K562 control cells treated with a combination of TKI and asciminib. These cells express much lower levels of ABCG2 compared with resistant cells (13% vs 97%) yet significant reductions in LD50^asciminib^ were observed in the presence of both imatinib and nilotinib. Thus, while TKI-mediated inhibition of ABCG2 remains a plausible explanation for the cooperation observed when cells are treated with asciminib:TKI combinations, it is also possible that the drugs synergistically inhibit Bcr-Abl kinase. Indeed, data from our laboratory investigating the effect of asciminib:TKI combination treatment in patient mononuclear cells have demonstrated that even low nanomolar concentrations of asciminib potentiate imatinib- and nilotinib-mediated inhibition of Bcr-Abl in patients predicted to respond poorly to TKI therapy [40]. Because imatinib and nilotinib bind the inactive conformation of Bcr-Abl [41] and asciminib locks Bcr-Abl in this conformation, we hypothesise that the additional of asciminib enhances TKI binding. Thus the simultaneous targeting of the myristate binding pocket as well as the ATP-binding site is more effective than targeting either site in isolation. Importantly, data from both primary CML cells and the data presented here support the use of asciminib in combination with imatinib and nilotinib in patients.

Due to the current lack of availability of asciminib resistant patient samples, we are unable to confirm increased transporter expression in primary leukemic cells at this time; only four patients worldwide have relapsed or harbor progressive disease, one due to confirmed myristate binding pocket mutations [42]. Importantly, there is precedence for alterations in transporter expression in response to therapy [28]. We have observed increased ABCB1 levels in patients receiving imatinib therapy thus increased ABCB1 and/or ABCG2 expression in response to asciminib remains a possibility.

In conclusion, asciminib resistant cell lines generated in this study demonstrated overexpression of ABCB1 and/or ABCG2 highlighting the key role of transporters in development of asciminib resistance. This is especially evident given that transporter expression was induced in two cell lines where basal expression levels are negligible to low. Susceptibility to ABCB1 overexpression is well recognised for imatinib and nilotinib resistance; indeed we have recently highlighted the importance in development of resistance [27] and potential for use of ABCB1 as a predictive biomarker of patient response [28]. We now show relevance of transporter overexpression in development of resistance to asciminib. Taken together, these results demonstrate that asciminib is transported by both ABCB1 and ABCG2 and is likely susceptible to resistance mediated by overexpression of these transporters. Importantly, resistance was reversed upon inhibition of both ABCB1 and ABCG2 with specific inhibitors (cyclosporine, verapamil, Ko143). Simultaneous administration of sub-efficacious concentrations of imatinib or nilotinib in combination with asciminib significantly enhanced asciminib efficacy in control cells and, to a greater extent, in resistant cells with ABCG2 overexpression in a synergistic manner. The data presented here provide an additional rationale for using imatinib or nilotinib in combination with asciminib, especially in the context of ABCG2 overexpression.

## MATERIALS AND METHODS

### Inhibitors

Imatinib mesylate (imatinib; Glivec; formerly STI-571), Nilotinib (Tasigna; formerly AMN107) and asciminib were provided by Novartis Pharmaceuticals (Basel, Switzerland). Inhibitors of ABCB1 and ABCG2 and preparation of stock solutions are detailed in Supplementary Materials and Methods.

### Cell lines

*BCR-ABL1*-expressing cell lines K562, KU812 (American Type Culture Collection (ATCC) Manassas, VA, USA) and the ABCB1 overexpressing variant, K562-Dox, (Leonie Ashman, University of Newcastle, Callaghan, NSW) were cultured as described previously [43]. K562-Dox cells stably overexpress ABCB1 as a result of long-term exposure of the parental K562 cell line to the ABCB1 substrate doxorubicin. K562-ABCG2 cells were generated from transduction of K562 cells with pcDNA3 vector containing full length ABCG2 as previously described [44]. In washout experiments, cells were washed 3× in drug free media, with a 30 min equilibration period between each wash, then cultured overnight before cell lysis and protein detection as described in Supplementary Materials and Methods.

### Generation of asciminib-resistant cell lines

Cell lines maintained in liquid culture were gradually exposed to escalating concentrations of asciminib over a 6 month time period [30]; parental control cell lines cultured in 0.1% DMSO were maintained in parallel. Asciminib was escalated once cells demonstrated >80% survival in culture for >10 days to a final concentration of 10 μM. This concentration is clinically unachievable based on the average peak steady state plasma levels in patients receiving 200 mg BID of ∼6.7 μM. Prior to all experimentation, cells were washed 3× in drug free media and left to equilibrate for 30 min in between each wash.

### Cell viability assays: LD50

Cells were resuspended in fresh culture media before culture in 24-well plates (Thermo Fisher Scientific, Waltham, MA, USA) in the presence of TKI or asciminib at a density of 2 × 10^5^ cells/mL. Plates were seeded with 1 mL of cell suspension and incubated for 72 h before cell viability determination with 7-aminoactinomycin (7-AAD; Invitrogen Life Technologies, Carlsbad, CA, USA) and Phycoerythrin (PE)-conjugated Annexin V (BD Biosciences, Franklin Lakes, NJ, USA). Flow cytometric analysis was conducted with a BD LSRFortessa^™^ X-20 (BD Biosciences) and FACSDiva^™^ software (BD Biosciences). The lethal dose of asciminib (LD50^asciminib^), imatinib (LD50^IM^), nilotinib (LD50^NIL^) and dasatinib (LD50^DAS^) required to cause 50% death of cells was calculated.

### Flow cytometry and fluorescent substrate efflux studies

ABCB1 and ABCG2 cell surface expression and function were measured as described previously [43]. Percentage expression was evaluated in comparison to isotype controls (Dakocytomation, Carpinteria, CA, USA). Flow cytometric analysis was conducted with a BD LSRFortessa™ X-20 (BD Biosciences) and FACSDiva^™^ software (BD Biosciences), post acquisition analysis was conducted with FlowJo v10.2 software (FlowJo LLC, Ashland, OR, USA).

### *BCR-ABL1* quantitation and mutation analysis

*BCR-ABL1* mRNA expression levels were quantitated using the TaqMan Universal PCR Master Mix (Applied Biosystems) and the ABI Prism 7500 Sequence Detection System (Applied Biosystems) as described previously [45]. *BCR-ABL1* kinase domain sequencing was performed as described previously [46]. Primer sequences used to sequence the myristate binding domain are available in Supplementary Materials and Methods.

### Western blotting for Bcr-Abl

Western blotting for total Bcr-Abl (1:1000; c-Abl Cell Signaling Technologies, Beverly, MA, USA), phosphorylated-Abl^Y245^ (1:1000; Cell Signaling Technologies) and phosphorylated-Bcr^Y177^ (1:1000; Cell Signaling Technologies) was performed using the BIO-RAD Trans-Blot^®^ Turbo™ Blotting System as detailed in Supplementary Materials and Methods.

### Milliplex^®^ MAP: Cell signaling multiplex assay

The Milliplex^®^ MAP kit (Merck Millipore) was used to determine the expression levels of p-STAT-5A/B. The assay was carried out in accordance with the manufacturer’s instructions using the Luminex MAGPIX^®^ instrument (Luminex Corporation, Austin TX, USA) and analyzed with xPONENT^®^ software (Luminex, version 4.2.1324.0).

### Statistics

Statistical tests were performed using the GraphPad Prism 5 statistical software (GraphPad Prism Inc, La Jolla, CA, USA). Normality tests were performed on each data set using the D’Agostino & Pearson omnibus normality test. The Mann-Whitney Rank Sum or the Student’s *t*-test were used to determine differences between experimental groups depending on whether the data sets failed or passed the normality test, respectively. Differences were considered to be statistically significant when the probability value (*p*-value) was <0.05. Combination indices were calculated with CalcuSyn software version 2.11 (Biosoft, Cambridge, United Kingdom).

## SUPPLEMENTARY MATERIALS FIGURES AND TABLE





## Abbreviations

TKI/s: Tyrosine Kinase Inhibitor/s
CML: Chronic Myeloid Leukemia
MFI: Mean Fluorescence Intensity
7-AAD: 7-aminoactinomycin
PE: Phycoerythrin
LD50: Lethal Dose 50%
