# Supplementary material for: The new allosteric inhibitor asciminib is susceptible to resistance mediated by ABCB1 and ABCG2 overexpression in vitro
Source: Oncotarget. 2018 Feb 3;9(17):13423–37. doi: 10.18632/oncotarget.24393 (PMC5862588; doi:10.18632/oncotarget.24393)
Supplement: Supplementary file 1 [file oncotarget-09-13423-s001.pdf]

The new allosteric inhibitor asciminib is susceptible to resistance mediated by ABCB1 and ABCG2 overexpression *in vitro*

SUPPLEMENTARY MATERIALS

Inhibitors

Stock solutions were prepared at 10 mM with distilled water and stored at –70° C (imatinib) or with DMSO and stored at 4° C (nilotinib, ABL001). Ko143 is an analogue of fumitremorgin C provided by Dr John Allen, Centenary Institute, Sydney, Australia and was used at 0.5 µM. 1 mM stock solutions and 100 µM working solutions were prepared in sterile water and stored at –80°C. Verapamil (RAH Pharmacy Adelaide, Australia) was used at 50 µM from a 2.5 mg/mL stock dissolved in H_2O. Cyclosporine A (Sigma-Aldrich, St. Louis, MO, USA) was used at 10 µM from a 4.15 mM stock prepared in DMSO. PSC-833 is a cyclosporine A derivative kindly provided by Novartis Pharmaceuticals and was used at 10 µM from 8.23 mM stock.

BCR-ABL1 quantitation and mutation analysis

A long PCR method was used to sequence the myristate binding domain using the following primers: Long2F 5′ ACT ATG AGC GTG CAG AGT GGA 3′ and Long2R 5′ GAG GGA GCA ATG GAG ACA CG 3′. A second-stage PCR used the forward primers: Seq1-220F 5′ CGC AAC AAG CCC ACT GTC T 3′ and Seq2-316F 5′ GAG TTC ATG ACC TAC GGG AAC CT 3′ and the reverse primer: Seq1-505R 5′ TTC GTC TGA GAT ACT GGA TTC CTG 3′.

Western blotting for Bcr-Abl

Following lysis in Igepal™ detergent (Sigma-Aldrich, St Louis, MO, USA), 40 µg protein was resolved on a BIO-RAD 4–15% Criterion™TGX™ Precast Gel and transferred to PVDF membrane using Midi Transfer Packs (BIO-RAD, Hercules, CA, USA) at 2.5 A for 10 min. Membranes were cut in half to allow separate probing of the control protein β-tubulin (1:1000, Cell Signalling Technologies). The top half was initially blocked in 5% Bovine Serum Albumin (BSA, Sigma-Aldrich) and probed with p-Abl^{Y245} overnight prior to incubation with alkaline-phosphatase (AP)-conjugated anti-rabbit immunoglobulin (Santa Cruz Biotechnology, Santa Cruz, CA, USA) and detection with ECF substrate (GE Healthcare) by FluorImager analysis (Molecular Dynamics, Sunnyvale, CA, USA). Membranes were stripped with Western Stripping Buffer (Alpha Diagnostic, San Antonio, TX, USA), re-blocked in 5% BSA, probed with c-Abl overnight, then AP-conjugated anti-rabbit immunoglobulin (Cell Signalling Technologies) and detected with ECF as above. Detected proteins were quantified using ImageStudioLite 5.0 software and expression normalized to control.

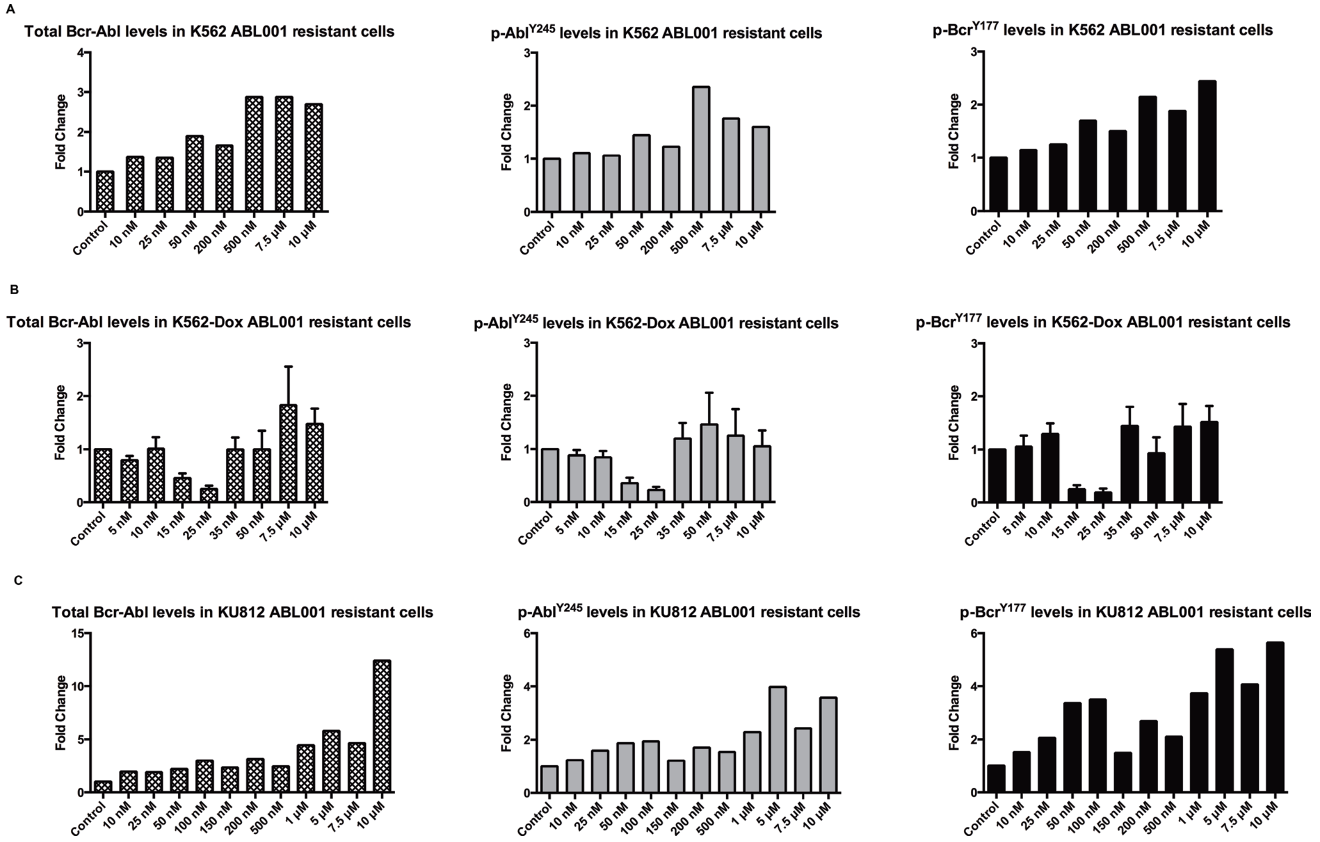

Supplementary Figure 1: ABL001 resistant K562 and KU812 cells demonstrate increased Bcr-Abl protein and activity levels. Expression levels of total and phospho-Bcr-Abl (Y177 and Y245) were assessed in ABL001 resistance intermediates of (**A**) K562 (**B**) K562-Dox and (**C**) KU812 cells. The densitometry analyses represent the mean of at least two separate western blots. Protein levels were normalised to β-tubulin control. Error bars represent SEM.

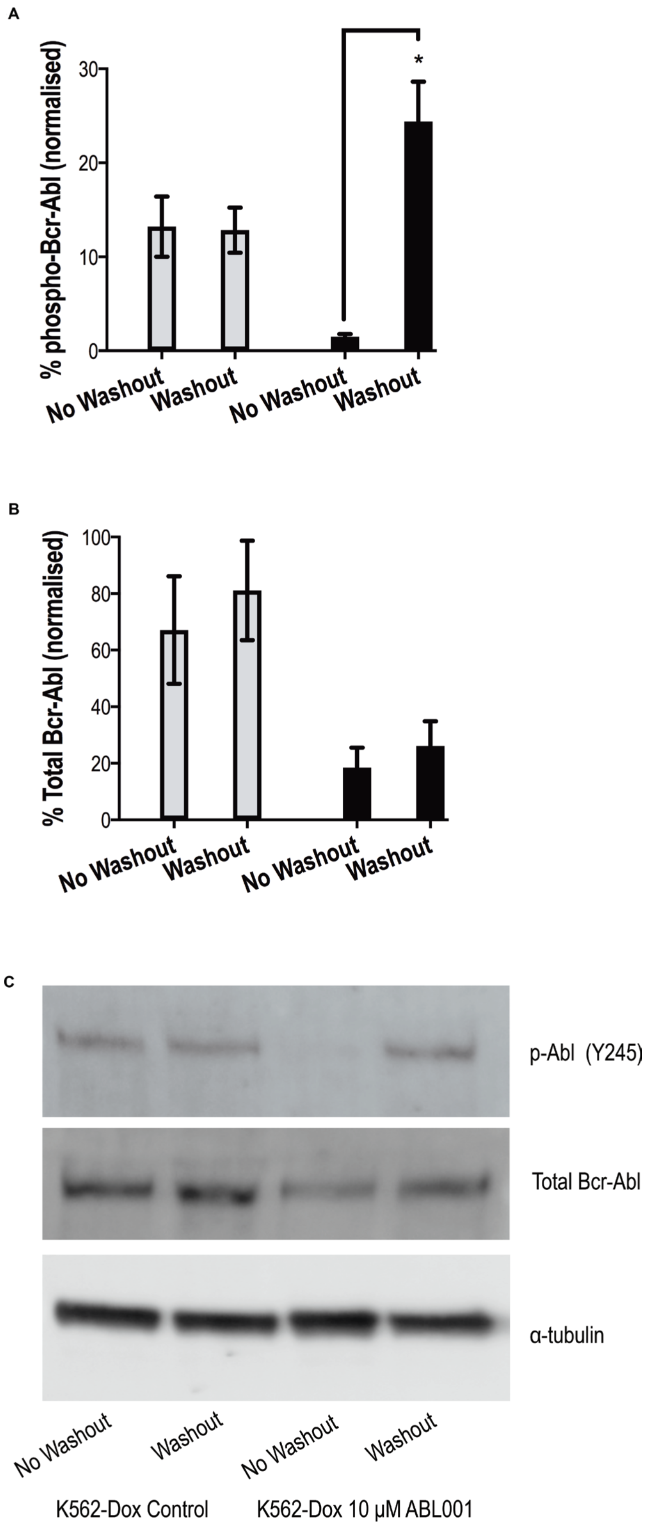

Supplementary Figure 2: ABL001 resistant K562-Dox cells demonstrate Bcr-Abl independent resistance. Expression levels of (**A**) phospho (Y245) and (**B**) total Bcr-Abl were assessed during development of nilotinib resistance in K562-Dox control cells and K562-Dox 10 μM ABL001 cells. (**C**) the western blot analyses are representative and the corresponding densitometry analysis represents the mean of three experiments. Protein levels were normalised to α-tubulin control. Statistical analyses were performed using Student's t-test with statistically significant *p*-values denoted by asterisks ($^{*}p < 0.05$). Error bars represent SEM.

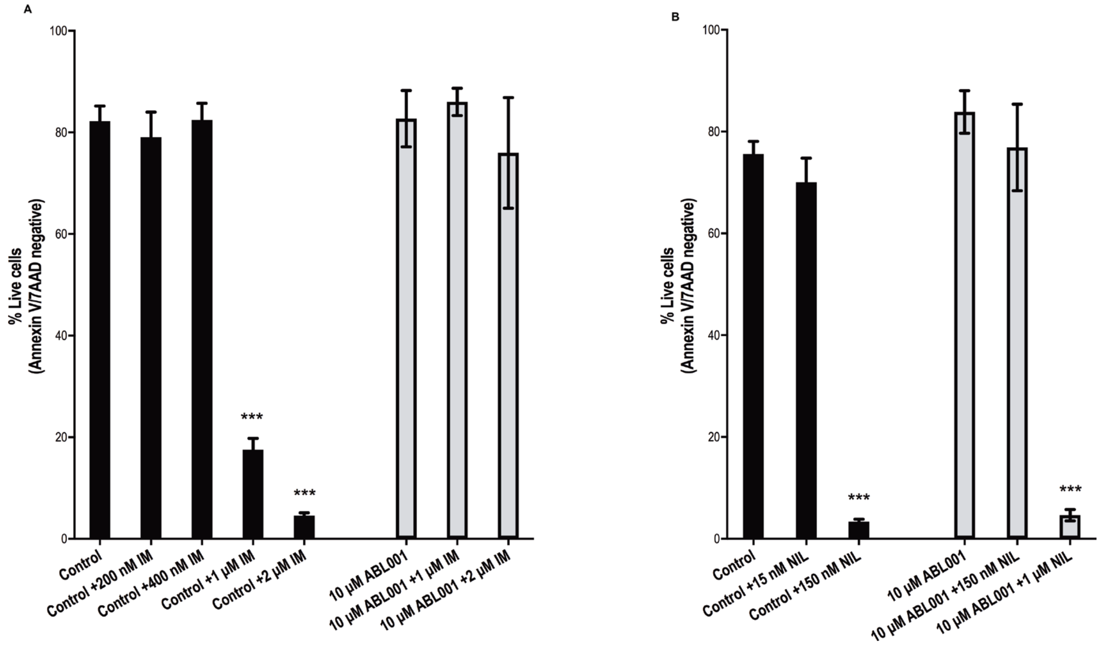

Supplementary Figure 3: 1 µM and 2 µM imatinib and 150 nM nilotinib do not cause significant cell death in K562 10 µM ABL001 cells when used as single agents. K562 control and K562 10 µM ABL001 cells were cultured for 72 h in the absence vs presence of (**A**) imatinib or (**B**) nilotinib and % live cells determined. Statistical analyses compared live cells in the presence of the indicated concentrations of TKI with live cells in the absence TKI. Data represent the mean of at least 3 independent experiments. Analyses were performed using unpaired Student's *t*-test (Welch's correction was applied for data groups with unequal SD). Statistically significant *p*-values are denoted by asterisks ($^{*}p < 0.05$, $^{**}p < 0.01$, $^{***}p < 0.001$). Error bars represent SEM. IM = imatinib. NIL = nilotinib.

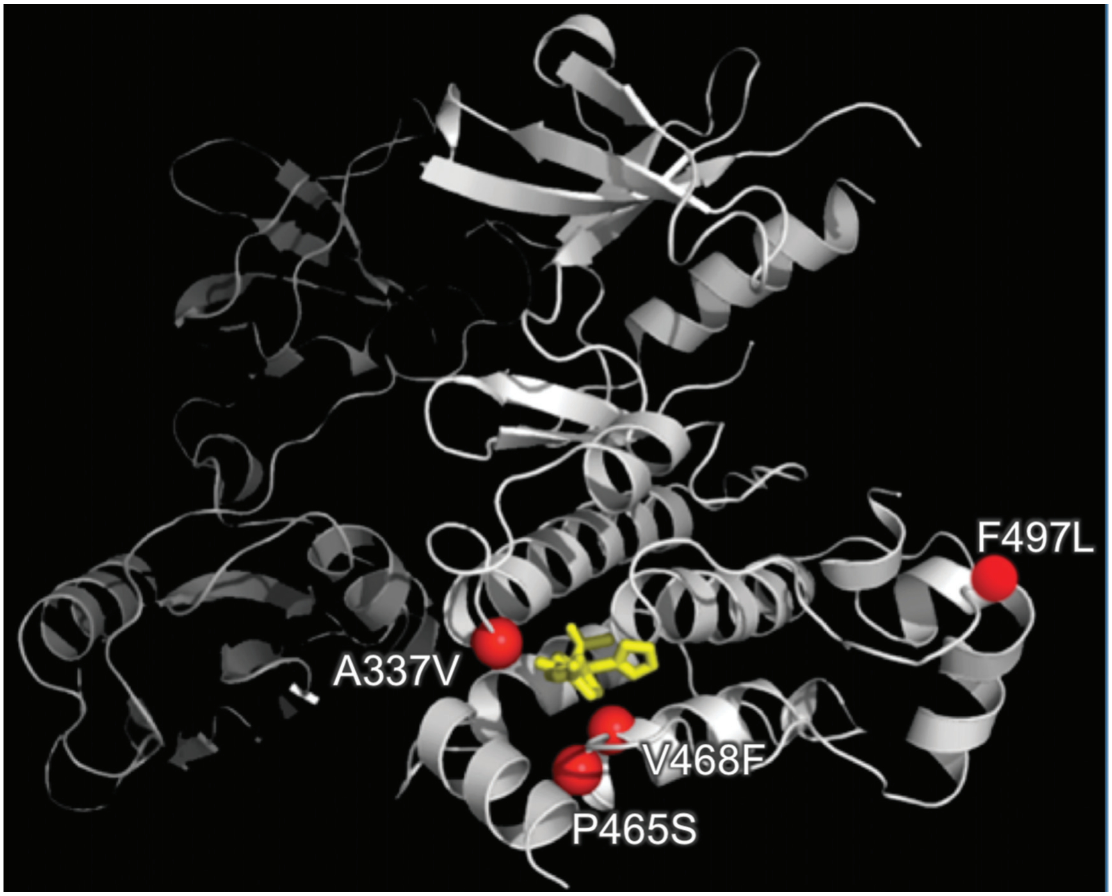

Supplementary Figure 4: The F497L myristate-binding pocket mutation is remotely located compared with known ABL001 resistant mutations (adapted from Ottman *et al.* [45]. A ribbon diagram of the X-ray crystal structure of the c-abl kinase domain (white ribbon) as well as SH2 and SH3 domains (grey ribbon). The myristate-binding pocket mutations known to be resistant to ABL001 and the newly identified F497L mutation are shown (red circles). β-sheets (arrows) and α-helices are also depicted and ABL001 is shown in yellow.

Supplementary Table 1: Combination indices for asciminib combined with TKIs as well as the dose effects of drugs alone.
See Supplementary_Table_1
